# Mac1-Dependent Copper Sensing Promotes *Histoplasma* Adaptation to the Phagosome during Adaptive Immunity

**DOI:** 10.1128/mbio.03773-21

**Published:** 2022-04-11

**Authors:** Stephanie C. Ray, Chad A. Rappleye

**Affiliations:** a Department of Microbiology, Ohio State University, Columbus, Ohio, USA; University of British Columbia

**Keywords:** Mac1, copper, transcription factor, *Histoplasma capsulatum*, pathogenesis, nutritional immunity, catalase, *Histoplasma*, macrophages

## Abstract

Intracellular pathogens residing within macrophage phagosomes are challenged with recognizing the phagosomal environment and appropriately responding to changing host defense strategies imposed in this organelle. One such phagocyte defense is the restriction of available copper as a form of nutritional immunity during the adaptive immune response to fungal pathogens. The intracellular fungal pathogen Histoplasma capsulatum adapts to this decreased copper through upregulation of the Ctr3 copper transporter. In this study, we show that *Histoplasma* recognizes the characteristic low-copper phagosomal environment of activated macrophages through the copper-dependent transcriptional regulator Mac1. Multiple *cis*-acting regulatory sequences in the *CTR3* promoter control upregulation of *CTR3* transcription under low-copper conditions, and the loss of Mac1 function prevents induction of Ctr3 under low-copper conditions. During adaptive immunity, this loss of copper sensing by Mac1 attenuates *Histoplasma* virulence more severely than loss of Ctr3 alone, indicating that Mac1 controls the expression of additional mechanisms important for pathogenesis. Transcriptional profiling of *Histoplasma* yeasts identified a small regulon of Mac1-dependent genes, with the most strongly regulated genes encoding proteins linked to copper, iron, and zinc homeostasis and defenses against reactive oxygen (iron-requiring catalase [CatB] and superoxide dismutase [Sod4]). Accordingly, the loss of Mac1 function increased sensitivity to copper and iron restriction and blocked low-copper-induced expression of extracellular catalase activity. Thus, Mac1-mediated sensing of low-copper signals to *Histoplasma* yeasts their residence within the activated macrophage phagosome, and in response, *Histoplasma* yeasts produce factors, including non-copper-dependent factors, to combat the enhanced defenses of activated macrophages.

## INTRODUCTION

Copper is an essential trace nutrient for virtually all cells, but it can be toxic in high concentrations. In fungi, copper is required as a metal cofactor for many enzymes, including the reactive oxygen-detoxifying superoxide dismutases, melanin biosynthetic enzymes, and mitochondrial cytochrome oxidase. However, excess copper is disruptive to iron-sulfur clusters and contributes to the production of damaging reactive oxygen species through Fenton reactions (1). Due to this dual nature of copper, copper acquisition in fungi has evolved to be tightly regulated. This regulation becomes especially important for proliferation within the host as fungal pathogens encounter both high- and low-copper environments during infection (2).

As an intracellular pathogen of host phagocytic cells, the dimorphic fungus Histoplasma capsulatum must acquire nutrients, including copper, to survive and replicate within a canonically microbicidal environment. The temperature-mediated transition to the pathogenic yeast phase defines much of *Histoplasma*’s virulence program (3, 4). Transcriptional analysis of avirulent *Histoplasma* mycelia compared to pathogenic yeasts confirms yeast-phase upregulation of virulence-associated genes, including alpha-glucan cell wall polysaccharides (5), internal and external catalases (6), the secreted Cu/Zn superoxide dismutase, Sod3 (7), and the high-affinity copper transporter, Ctr3 (8). The Ryp family of transcription factors are required for the transition to the yeast phase and control 96% of yeast-phase gene expression, allowing for simultaneous upregulation of these virulence factors upon exposure to mammalian body temperature (9, 10). The linkage of many virulence genes to the temperature-driven yeast phase suggests this phase can in some regards be considered “programmed” for virulence in mammalian hosts (3, 11).

Mammalian body temperature is a crucial primary indicator of the host environment for *Histoplasma*, but the mammalian host is not a static environment over the course of an infection. As such, temperature alone is insufficient to signal all conditions and the ensuing responses of *Histoplasma* for pathogenesis. For example, yeasts which lack the Ctr3 copper transporter are as virulent as wild-type *Histoplasma* during the innate immune stage of infection (8). However, during the adaptive immune response, Ctr3 is required for full virulence (8). Transcription of the *CTR3* gene is stimulated not only by the transition from the avirulent mycelia to the virulent yeast phase but also by the limitation of available copper (8). Consistent with this, treatment of macrophages with interferon gamma (IFN-γ), which activates macrophages and induces phagosomal copper restriction, results in an increase in *CTR3* expression by intracellular *Histoplasma* yeasts (8). Since copper availability signals changes to the macrophage phagosome during infection and can influence the proliferation of *Histoplasma* within macrophages, we investigated the mechanism underlying copper sensing by intracellular yeasts. Our findings indicate that in a copper-restricted environment, the copper-dependent transcription factor Mac1 induces expression of not only Ctr3 but also additional factors with potential virulence mechanisms beyond copper acquisition.

## RESULTS

### Upregulation of the *CTR3* copper transporter gene in low copper requires promoter-localized copper response elements.

**FIG 1:**
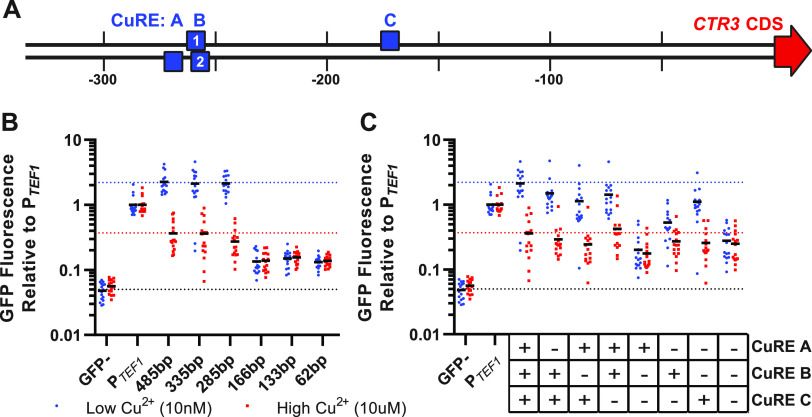
Upregulation of the *CTR3* minimal promoter in low copper requires MAC1 binding motifs. (A) Schematic of the Histoplasma capsulatum minimal *CTR3* promoter, with copper response elements (CuREs) A, B1 B2, and C denoted by blue boxes. Due to a significant overlap between B1 and B2, these were treated as one site (“CuRE B”) for promoter mutation experiments. (B) GFP fluorescence driven by sequentially truncated *CTR3* promoters in low (20 nM, blue circles) and high (5 μM, red squares) copper, shown relative to constitutively expressed GFP (P*_TEF1_*). (C) GFP fluorescence driven by the minimal 335-bp *CTR3* promoter in the presence or absence of CuREs A, B, and C in low (20 nM, blue circles) and high (5 μM, red squares) copper, shown relative to constitutively expressed GFP (P*_TEF1_*). The GFP^–^ strain G217B is included in panels B and C as a reference for background levels of fluorescence. The dotted lines in panels B and C represent wild-type levels of GFP fluorescence in low copper (blue), high copper (red), and background fluorescence in the GFP^–^ strain G217B (black). For panels B and C, *n* = 16 biological replicates for each strain were replica plated on high- and low-copper media.

To identify the *cis*-acting regulatory elements necessary for the upregulation of *CTR3* under low-copper conditions, we first determined the minimal *CTR3* promoter which retains transcriptional regulation by copper. To define this minimal promoter, we fused sequential truncations of the region upstream of the *CTR3* coding region to a green fluorescent protein (GFP) reporter gene. These transcriptional reporters, consisting of 485, 335, 285, 166, 133, and 62 bp upstream of the *CTR3* start codon, were transformed into *Histoplasma* yeasts and the GFP fluorescence measured under low-copper (20 nM CuSO_4_) and high-copper (5 μM CuSO_4_) conditions (Fig. 1A and B). GFP reporter gene fluorescence driven by the *CTR3* promoter truncations was normalized to the constitutively expressed *TEF1* promoter (P*_TEF1_*) to control for any copper-dependent differences in growth or general transcription. A GFP-negative control (strain G217B) was also included to indicate background levels of yeast fluorescence (Fig. 1B). The 485-, 335-, and 285-bp *CTR3* promoters showed consistent levels of robust fluorescence in low copper and decreased fluorescence in high copper similar to what was previously quantified by qRT-PCR (8), indicating copper-dependent regulation of *CTR3* transcription. However, upstream regions less than 285 bp resulted not just in the loss of copper-dependent regulation of transcription but also in the significant loss of GFP reporter fluorescence below the level of *CTR3* transcription even under repressed conditions (i.e., high copper). Therefore, the 285-bp promoter was defined as the minimal *CTR3* promoter for further experiments on transcriptional regulation.

The results of the *CTR3* promoter truncations focused attention on potential regulatory elements within the 285-bp upstream region. Since copper regulates *CTR3* expression, we used the defined S. cerevisiae copper-response element (CuRE) motif to search for the presence of potential copper-response elements within the 285-bp *Histoplasma CTR3* promoter region. Three putative CuREs were identified within the *CTR3* promoter at bases −273, −260, and −176 relative to the *CTR3* start codon and were designated A, B_2_, and C, respectively. A fourth putative CuRE (designated B_1_) was manually identified as overlapping with site B_2_ at bp −263. CuREs A and B2 were on the strand opposite *CTR3* transcription, whereas CuREs B1 and C were on the sense strand (Fig. 1A). To determine whether these putative CuREs functioned in the regulation of the *CTR3* promoter by copper, we scrambled the nucleotide sequences of these sites individually or in combination within the 335-bp *CTR3* promoter-GFP transcriptional reporter (see Fig. S1 in the supplemental material). Scrambling the sequence of individual CuREs caused a modest decrease in GFP fluorescence in low copper, indicating that all putative CuREs are necessary for the full copper regulation of CTR3 transcription (Fig. 1C). Due to their overlap, CuRE B1 and B2 could not be individually disrupted and were considered a single site (“B”) for the analysis. Analysis of fluorescence when only a single CuRE remained unchanged showed that CuRE C alone was sufficient for retention of significant copper-dependent regulation, albeit not to the full wild-type *CTR3* transcription levels. Loss of all three CuREs abolished the copper regulation similar to truncation of the *CTR3* promoter to <285 bp, indicating that the upregulation of *CTR3* in low copper requires multiple CuRE motifs in the *CTR3* promoter located within the 285-bp upstream region.

10.1128/mbio.03773-21.3 FIG S1 *CTR3* minimal promoter sequence with identified motifs for copper-regulation. Sequence of the minimal *CTR3* promoter from Histoplasma capsulatum strain G217B. Boxes denote putative Mac1 binding sites as identified via FIMO (solid lines) and manually (dotted lines). Native and scrambled sequences are represented by black and blue text, respectively. Red arrow denotes the start codon of the *CTR3* coding sequence (CDS). The Saccharomyces cerevisiae Mac1 binding motif (CuRE, JASPAR matrix ID MA0326.1) is included at top left. Download FIG S1, EPS file, 2.8 MB. Copyright © 2022 Ray and Rappleye. 2022 Ray and Rappleye. https://creativecommons.org/licenses/by/4.0/ This content is distributed under the terms of the Creative Commons Attribution 4.0 International license.

### The Mac1 transcription factor regulates expression of *CTR3* and the ability to grow in limited copper.

**FIG 2:**
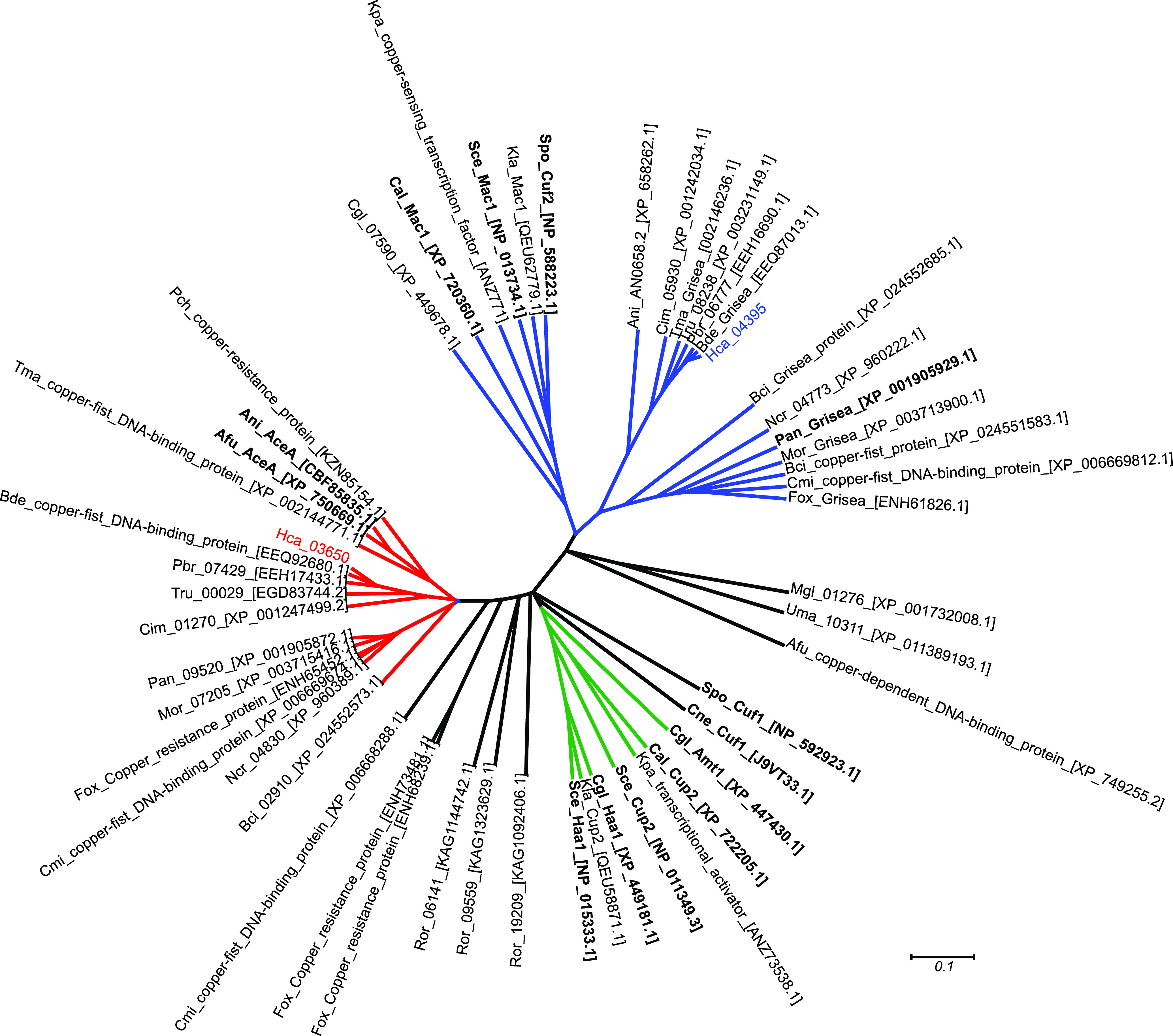
Phylogenetic relationships of fungal copper-fist domain-containing proteins. The amino acid sequence of the copper-fist domain in fungal proteins was aligned and used to construct a phylogenetic tree to infer relatedness among proteins. A monophyletic clade of Mac1/Grisea orthologs (blue branches) include a putative *Histoplasma* Mac1 protein (Hca_04395). The other *Histoplasma* copper-fist protein (Hca_03650) groups with AceI/AceA orthologs (red clade). AceI/Cup2 and AceI-paralogs in hemiascomycetes are indicated by green branches. Labels indicate fungal proteins from Aspergillus fumigatus (Afu), Aspergillus nidulans (Ani), Botrytis cinerea (Bci), Blastomyces dermatitidis (Bde), Candida albicans (Cal), Candida glabrata (Cgl), *Coccidioides immitis* (Cim), Cordyceps militaris (Cmi), Cryptococcus neoformans (Cne), Fusarium oxysporum (Fox), Histoplasma capsulatum (Hca), Kluyveromyces lactis (Kla), *Komagataella pastoris* (Kpa), *Malassezia globose* (Mgl), Magnaporthe oryzae (Mor), Neurospora crassa (Ncr), Podospora anserina (Pan), Paracoccidioides brasiliensis (Pbr), Penicillium chrysogenum (Pch), *Rhizopus oryzae* (Ror), Saccharomyces cerevisiae (Sce), Schizosaccharomyces pombe (Spo), *Talaromyces marneffei* (Tma), Trichophyton rubrum (Tru), and Ustilago maydis (Uma), with accession numbers indicated in brackets. Proteins with experimental evidence of roles in copper response/homeostasis are indicated in boldface.

In S. cerevisiae, CuRE motifs are bound by the Mac1 transcription factor (12–14). Multiple copper-dependent transcription factors exist in fungi, including Mac1, AceI/Cup2/Amt1, and Cuf1. Each is characterized by the presence of a copper-fist domain (pfam00649). The Grisea protein, originally named after a gray-pigmented mutant of Podospora anserina that does not produce the multicopper oxidase laccase (15), represents a Mac1-like copper-dependent transcription factor (16). In many fungi, Mac1/Grisea induces transcription of genes in low copper, whereas AceI homologs tend to control expression of genes in high copper environments (17). However, some fungi have a single protein (e.g., Cryptococcus Cuf1) that appears to be a hybrid between AceI and Mac1 and modulates transcriptional responses to both high and low copper (18). To determine which potential copper-dependent transcription factors are encoded in the *Histoplasma* genome, proteins with a copper-fist domain and homologs of the well-studied Mac1, Cup2, and Cuf1 among diverse fungi were grouped by phylogenetic relatedness (Fig. 2). Most euascomycete fungi encode one ortholog of Mac1 and a second AceI/Cup2-like ortholog. Many hemiascomycete fungi have two AceI/Cup2-like proteins (e.g., Cup2 and Haa1 of *Saccharomyces*) within a clade distinct from the Mac1 orthologs. Notable exceptions to the dual copper-responsive transcription factor situation are fungi that only have a single copper-fist domain protein (e.g., Basidiomycetes Cryptococcus, *Malassezia*, and *Ustilago*). A few fungi (e.g., *Rhizopus oryzae*, Cordyceps militaris, and Fusarium oxysporum) have at least three copper-fist domain-containing proteins, although the reason for the expansion of these proteins is unknown. The *Histoplasma* genome encodes a single Mac1 ortholog (Hca_04395) and an AceI/Cup2 ortholog (Hca_03650), and this dual copper-fist regulator situation is present in related dimorphic fungi (*Blastomyces*, *Paracoccidioides*, and *Coccidioides*).

**FIG 3:**
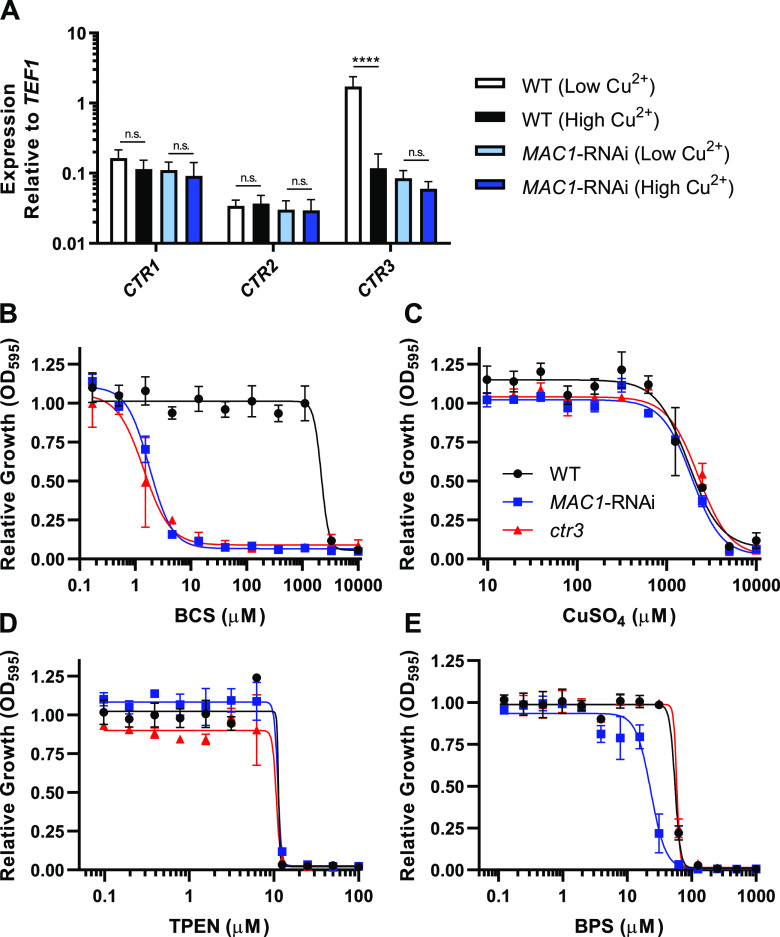
*MAC1* knockdown leads to loss of *CTR3* induction and sensitivity to low copper. (A) Gene expression of H. capsulatum *CTR1*, *CTR2*, and *CTR3* in wild-type (white/black bars) versus *MAC1*-depleted (blue bars) yeasts grown in low copper (20 nM, lighter bars) versus high copper (5 μM, darker bars). Expression is reported relative to *TEF1*. (B to E) Growth of wild-type (black circles), *MAC1*-depleted (blue squares), and *ctr3* mutant (red triangles) yeasts on a concentration gradient of copper chelator BCS (B), excess CuSO_4_ (C), zinc chelator TPEN (D), and iron chelator BPS (E). Growth in panels B to E is reported relative to growth in untreated media. Error bars denote the standard deviations from the mean. *P* values were calculated using Student’s t-test (****, *P* < 0.0001; n.s., not significant).

To determine whether the *Histoplasma* Mac1 protein regulates expression of the *CTR* family of copper transporter-encoding genes, low-copper induction of *CTR* gene transcription was surveyed following depletion of Mac1. Mac1 was depleted from *Histoplasma* yeasts by RNAi (5), and the yeasts were grown in either high copper (5 μM CuSO_4_) or low copper (no added Cu; ∼20 nM). Expression of the *CTR1*, *CTR2*, and *CTR3* genes was quantified via qRT-PCR. Depletion of Mac1 had no significant effect on expression of *CTR1* and *CTR2* (Fig. 3A). In Mac1-expressing yeast, low copper stimulated a 14-fold increase in *CTR3* expression compared to high-copper conditions, but this induction of transcription was absent in Mac1-depleted yeasts (Fig. 3A). *CTR3* expression in low copper in the absence of Mac1 resembled that of *CTR3* expression in high copper in Mac1-expressing cells. Therefore, *Histoplasma* Mac1 is required specifically for the upregulation of *CTR3* in low-copper conditions, which is consistent with the Mac1-dependent regulation of copper-related genes in other fungi (16, 19–21).

To determine whether Mac1 is involved in homeostasis of other metal ions, we tested the sensitivity of *Histoplasma* yeasts to various metal stresses in the absence of Mac1 function. Loss of Ctr3 function severely limits growth of *Histoplasma* in limiting copper (8). As expected, given the loss of *CTR3* expression in the *MAC1* knockdown strain, loss of Mac1 function decreased the 50% inhibitory concentration (IC_50_) of the copper-chelator BCS by >1,000-fold compared to wild-type *Histoplasma* yeasts (Fig. 3B). This sensitivity was nearly identical to that caused by loss of Ctr3, indicating that Mac1-dependent regulation of Ctr3 controls *Histoplasma*’s ability to grow in limiting copper. In contrast, the *MAC1* knockdown strain showed no defect in resistance to excess copper (Fig. 3C). There was also no change in the IC_50_ for the zinc chelator TPEN when Mac1 was depleted (Fig. 3D). Interestingly, loss of Mac1 function resulted in a modest (2-fold) decrease in the IC_50_ for the iron-specific chelator BPS compared to the IC_50_ for both wild-type yeasts and Ctr3-deficient yeasts, suggesting a slight decrease in the ability of the *MAC1* knockdown strain to scavenge iron (Fig. 3E).

### *Histoplasma* Mac1 is required for fungal virulence during the adaptive immune response.

**FIG 4:**
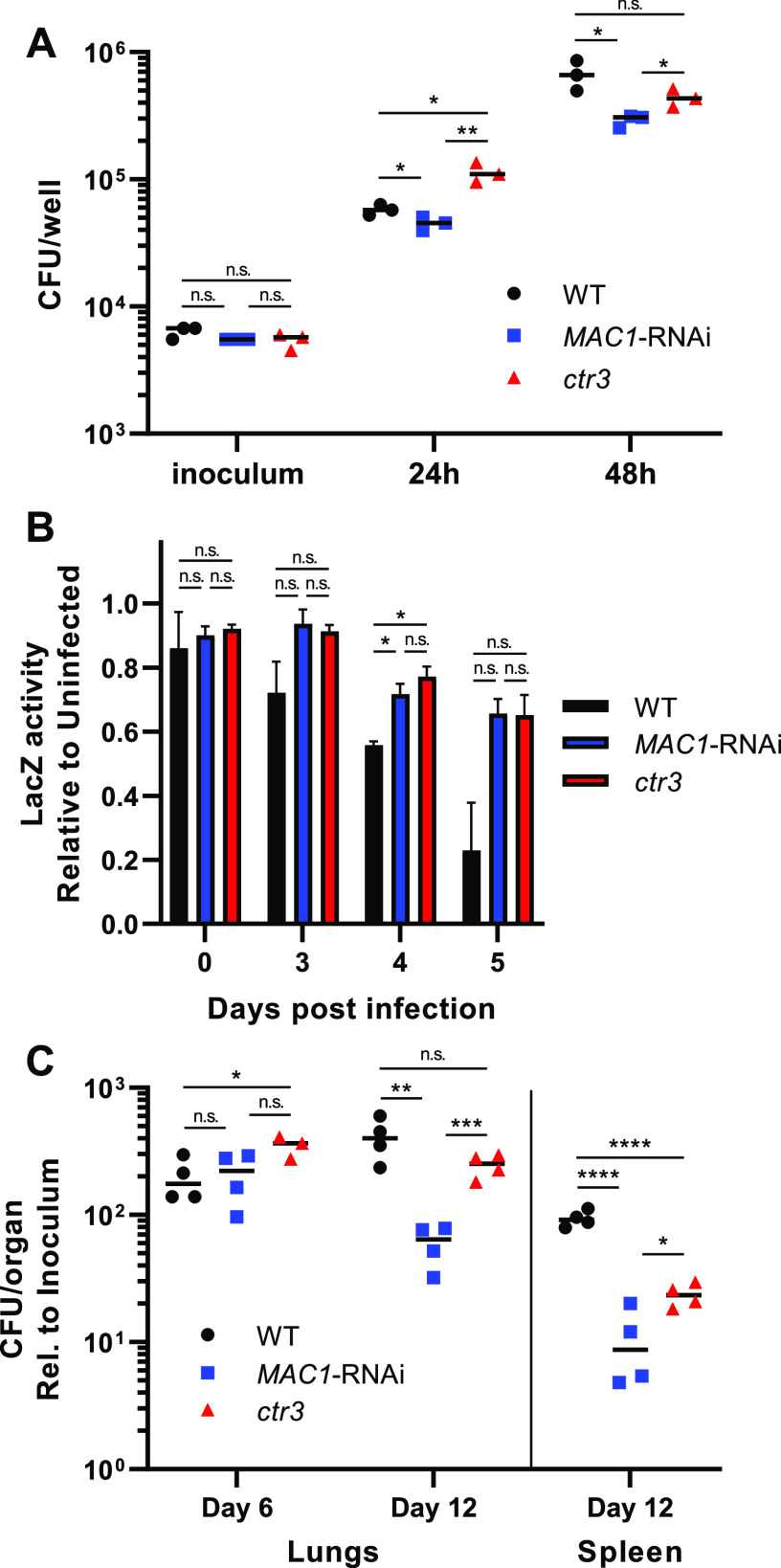
*MAC1* knockdown leads to attenuated virulence *in vitro* and *in vivo*. (A) CFU from macrophages infected with wild type (black circles), *MAC1*-depleted (blue squares), and *ctr3* mutant (red triangles) *Histoplasma* yeasts. (B) Macrophage-associated LacZ activity after infection with wild-type (black bars), *MAC1*-depleted (blue bars), and *ctr3* mutant (red bars) *Histoplasma*, shown relative to uninfected macrophages. (C) *Histoplasma* CFU recovered from murine lungs and spleens following intranasal infection with wild-type (black circles), *MAC1*-depleted (blue squares), and *ctr3* mutant (red triangles) yeasts. CFU are shown relative to the inoculum for each strain. Error bars denote the standard deviations from the mean. *P* values compared to wild-type yeast were calculated using Student’s t-test (*, *P* < 0.05; **, *P* < 0.01; ***, *P* < 0.001; ****, *P* < 0.0001; n.s., not significant).

Given the role of Mac1 in regulating *Histoplasma* acquisition of copper, the Mac1-depleted strain was tested for its ability to infect and proliferate in macrophages. Intracellular proliferation of *Histoplasma* yeasts was measured following infection of cultured macrophages. By 24 h postinfection, the Mac1-deficient strain showed reduced intracellular proliferation compared to wild-type yeasts (Fig. 4A). At 48 h postinfection, Mac1-depletion reduced intracellular proliferation to a greater degree than loss of Ctr3, suggesting that Mac1 controls aspects of virulence beyond Ctr3-mediated acquisition of copper. As a consequence of impaired intracellular proliferation, the Mac1-deficient and Ctr3-deficient strains were also attenuated in their ability to kill host macrophages (Fig. 4B).

Given the importance of copper acquisition to intracellular *Histoplasma* growth and the role of Mac1 in sensing available copper, we tested whether Mac1 was necessary for *Histoplasma* virulence *in vivo*. Respiratory infections were established in mice with wild-type, the Mac1-depleted, and the Ctr3-deficient mutant strains. At 6 days postinfection, neither the Mac1-depleted nor the Ctr3-deficient strains were attenuated compared to wild-type *Histoplasma* (Fig. 4C). This is consistent with our previous study (8) that indicated a copper-replete intracellular host environment during the innate immune stage before the onset of significant production of IFN-γ (22). At 12 days postinfection, after the onset of adaptive immunity, deficiency of either Mac1 or Ctr3 attenuated the virulence of *Histoplasma* in both respiratory and disseminated infections (fungal burden in lungs and spleens, respectively; Fig. 4C). Depletion of Mac1 resulted in greater attenuation than the loss of Ctr3 during lung infection (∼4-fold lower fungal burdens for the Mac1-depleted strain), but this difference was not as pronounced in the spleen (nearly 2-fold; Fig. 4C). These data confirm that Mac1-dependent regulation of Ctr3 is necessary for copper acquisition by *Histoplasma* yeasts when copper becomes limited during the adaptive immune response and also suggest that Mac1 regulates genes important for virulence in addition to *CTR3*.

### *Histoplasma* Mac1 regulates genes linked with metal homeostasis and ROS detoxification.

**FIG 5:**
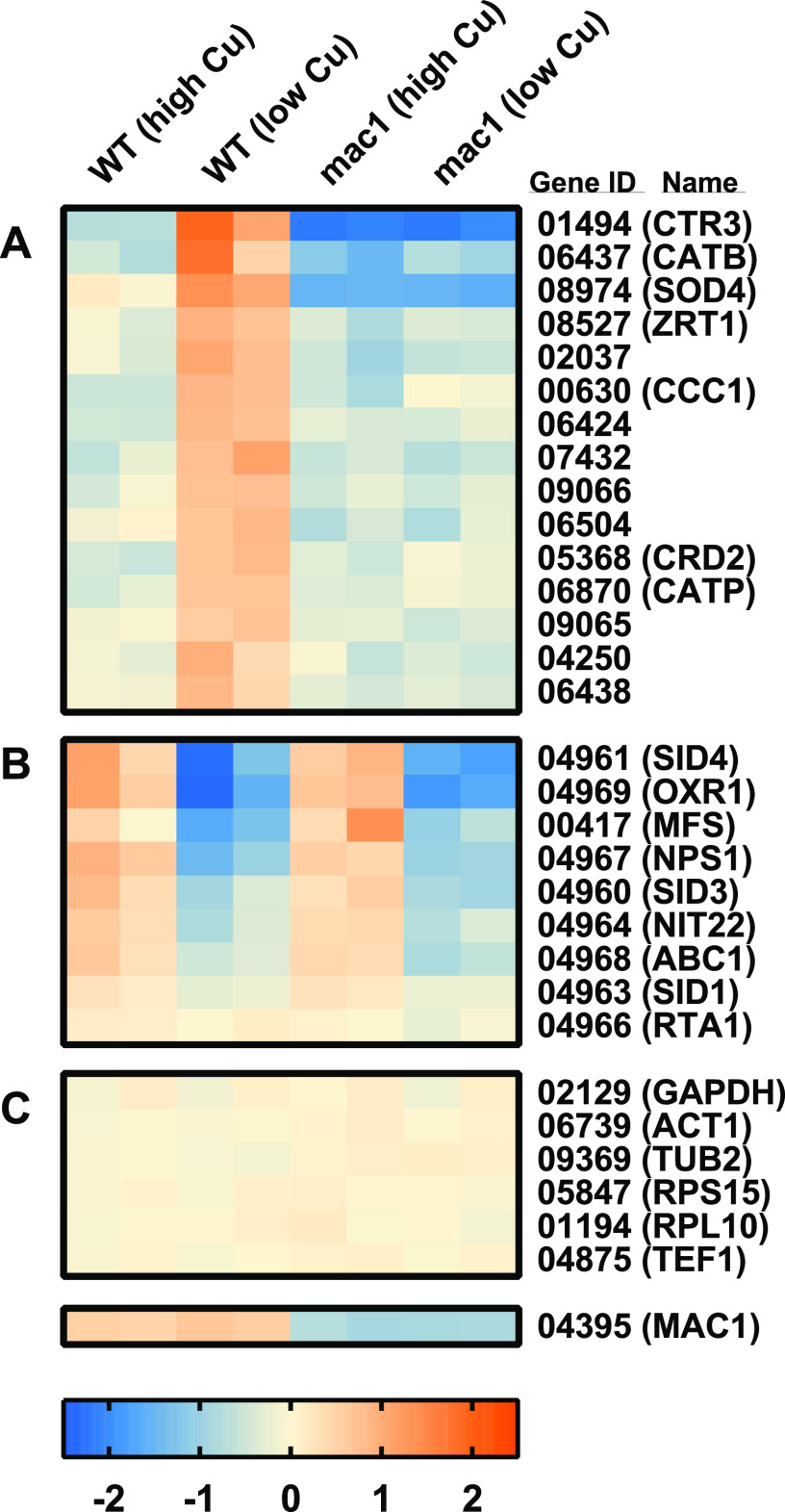
Mac1 regulates genes involved in metal acquisition and ROS detoxification. Transcriptional profiling of wild-type (WT) and *MAC1*-depleted (mac1) *Histoplasma* in high-copper (5 μM) and low-copper (20 nM) conditions, with color bars representing the log_2_-fold change under each condition compared to the average expression for each gene (red, increased expression; blue, decreased expression). Selected genes illustrate *MAC1*-dependent copper regulation (A), *MAC1*-independent copper regulation (B), and constitutively expressed genes (C). Each column represents data from one biological replicate (*n* = 2 for each condition).

To define the *Histoplasma* Mac1 regulon, we examined the transcriptomes of *Histoplasma* yeasts grown under copper-replete and copper-limited conditions, as well as in the presence or absence of Mac1. RNA was harvested from yeasts grown in a rich medium with either low-copper (∼20 nM trace copper) or high-copper (5 μM CuSO_4_) conditions, which are sufficient for up- and downregulation of *CTR3* expression (8). Twenty-three genes showed differential gene expression dependent on the amount of available copper (Fig. 5). Of these copper-regulated genes, differential gene expression analysis of wild-type versus Mac1-depleted yeasts revealed 15 genes were dependent on Mac1 function (Fig. 5A). The copper-regulated genes whose expression was independent of Mac1 (Fig. 5B) were 7 of the 8 genes of the putative siderophore biosynthesis gene cluster (23). Interestingly, these Mac1-independent siderophore synthesis genes were downregulated by low copper, opposite that of the Mac1-regulated genes. Within the Mac1 regulon, the gene with the highest differential expression was *CTR3*, which is consistent with earlier findings (Fig. 3A) and confirms the validity of the transcriptome data sets. Mac1 also regulates *CRD2*, a gene encoding a putative copper-binding factor related to copper homeostasis and the *CCC1* gene homologous to vacuolar metal ion transporters. Surprisingly, the Mac1 regulon includes many genes unrelated to copper homeostasis. Notable among these are *ZRT1*, a putative zinc transporter related to the Zrt2 zinc transporter (24); *CATB* and *CATP*, two catalases required for detoxification of host ROS (6); and *SOD4*, a predicted superoxide dismutase. Interestingly, the catalases are iron-, not copper-containing enzymes, and Sod4 is homologous to iron/manganese-type, not copper/zinc-type, superoxide dismutases. Various levels of copper or Mac1 do not affect general transcription since the expression of multiple housekeeping genes is not affected by copper availability or depletion of Mac1 (Fig. 5C).

### Extracellular catalase activity requires Mac1.

**FIG 6:**
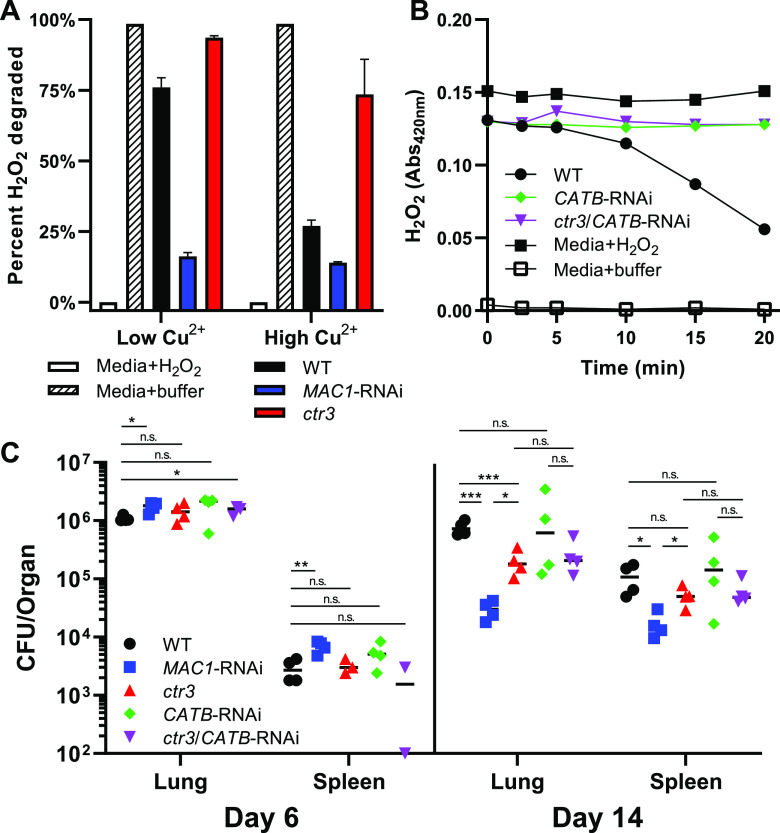
Extracellular catalase activity requires Mac1 but is dispensable for virulence. (A) Catalase activity of *Histoplasma* supernatants from wild-type (black bars), *MAC1*-deficient (blue bars; two independent strains), and *ctr3* mutant yeasts after a 20-min incubation with H_2_O_2_. Results for media with H_2_O_2_ (white bars) and media with buffer alone (hashed bars) are shown for comparison. (B) H_2_O_2_ degradation over time by wild-type (black circles), *CATB*-depleted (green diamonds), and *ctr3/CATB*-deficient (purple inverted triangles) *Histoplasma* yeast supernatants. Closed and open black squares denote H_2_O_2_ levels in media with added H_2_O_2_ and buffer alone, respectively. (C) *Histoplasma* CFU recovered from murine lungs and spleens following intranasal infection with wild-type (black circles), *MAC1*-depleted (blue squares), *ctr3* mutant (red triangles), *CATB*-depleted (green diamonds), and *ctr3/CATB*-deficient (purple inverted triangles) yeasts. Error bars denote the standard deviations from the mean. *P* values compared to wild-type yeast (except where another comparison is specified) were calculated using Student’s t-test (*, *P* < 0.05; **, *P* < 0.01; ***, *P* < 0.001; n.s., not significant).

Given that the virulence of yeasts that have lost Mac1 function is more attenuated than the virulence of Ctr3-deficient yeasts, we examined the contribution of Mac1-dependent expression of the CatB catalase, which has been linked to virulence (6). To validate the Mac1 regulation of the extracellular catalase-encoding *CATB* gene, we measured the catalase activity in yeast culture supernatants of wild-type, Mac1-deficient, and Ctr3-deficient yeasts. Consistent with the transcriptional data, supernatants from wild-type cultures grown in low copper exhibited nearly 3-fold more extracellular catalase activity than wild-type supernatants grown in excess copper (Fig. 6A). The Ctr3-deficient culture supernatants mirrored the catalase activity of the wild-type samples, confirming that the catalase activity is independent of copper acquisition. Supernatants from Mac1-deficient yeast lacked significant extracellular catalase activity in both low- and high-copper growth conditions. Decreased catalase production by Mac1-depleted yeasts in low copper was similar to that from wild-type yeasts grown in high copper, indicating the cause was failure to upregulate *CATB* expression in low-copper environments.

To test whether Mac1-dependent *CATB* expression accounts for the difference in virulence between the Mac1-depleted and *ctr3Δ* mutant strains, we depleted CatB from the Ctr3-deficient mutant. Depletion of CatB by RNAi eliminated the extracellular catalase activity of both wild-type and Ctr3-deficient mutant strains (Fig. 6B). These strains were used to infect mice, and fungal burdens were measured at early and late time points to determine whether the loss of CatB function, in addition to the Ctr3 loss, mimicked that of Mac1-depleted yeasts. At 6 days postinfection, the fungal burdens were similar for all of the strains tested (Fig. 6C), reflecting a copper-replete intracellular environment for *Histoplasma* before the adaptive immune stage and consequent lack of induction of *CTR3* and *CATB*. At 14 days postinfection, the loss of Mac1 and Ctr3 resulted in attenuated virulence, with Mac1-deficient yeasts showing a 6.7-fold reduction in fungal burden in the lungs and a 3.6-fold decrease in the spleen compared to the Ctr3-deficient mutant (Fig. 6C). The additional loss of CatB function in the Ctr3-deficient strain caused no further attenuation in the fungal burdens in the lungs or spleens than the loss of Ctr3. Therefore, the decreased virulence of the Mac1-depleted strain compared to the *ctr3Δ* mutant is not caused by a loss of Mac1-dependent upregulation of the extracellular catalase CatB.

## DISCUSSION

The regulation of gene expression in response to environmental conditions often includes both *cis*- and *trans*-acting components. Typically, this involves the binding of specific transcription factors to sequence motifs within the promoter of target genes. In this study, we define the *cis*- and *trans*-acting elements influencing the expression of the Ctr3 copper transporter of *Histoplasma*. We show that Mac1 is the transcription factor that requires copper-response element (CuRE) motifs within the promoter region of the Ctr3-encoding gene to promote *CTR3* transcription under low copper availability. In this regard, *Histoplasma* Mac1 is similar to Mac1 orthologs in other Ascomycetes, and the required CuREs present within the *Histoplasma CTR3* promoter are similar to the CuRE consensus motif defined for S. cerevisiae (12, 14) (Fig. 1), suggesting conservation of the mechanism for sensing of and response to various copper levels. However, unlike the situation in other fungi, the expression of *Histoplasma CTR3* is also controlled by morphology-regulating transcription factors such as Ryp1 (8). The *CTR3* promoter has motifs within the minimal 285-bp promoter that match the defined Ryp1-binding motifs, and the *CTR3* gene was found within the Ryp1 regulon, as defined by ChIP-seq (10). While the Ryp1-dependent regulation contributes to baseline expression of *CTR3* in yeasts (i.e., baseline expression in response to mammalian body temperature), Mac1 is required for the induction of *CTR3* when copper becomes limiting as a part of the mammalian immune response to infection. Together, the actions of Ryp1 and Mac1 contribute to activation of the *Histoplasma* pathogenesis program, as well as adaptation to changing phagosomal conditions due to activation of the host immune response.

While Mac1 is essential for expression of the Ctr3 virulence factor, Mac1-dependent regulation of additional genes also contributes to *Histoplasma* pathogenesis. *In vivo*, the contribution of non-CTR Mac1-regulated genes to *Histoplasma* virulence is specific for *Histoplasma* proliferation within phagocytes during the adaptive immune response (Fig. 4), just as it is for Ctr3 (8). Consistent with this, RNA-seq analysis shows Mac1 controls the upregulation of 15 genes in low-copper conditions, including *CTR3*. In comparison, the fungal pathogens Candida albicans and Cryptococcus spp. are only transiently intracellular and infect a wide range of host extracellular niches. Notably, the C. albicans Mac1 and C. neoformans Cuf1 regulons are much larger in these fungi, regulating 61 and 512 genes in response to low copper, respectively (25, 26). Furthermore, the A. fumigatus Mac1 homolog regulates 700 genes in A. fumigatus hyphae, which are extracellular during infection (27). Together, this suggests that the narrow Mac1 regulon in *Histoplasma* represents a highly tailored response to the near-exclusively intracellular lifestyle of *Histoplasma* yeasts.

Among the *Histoplasma* Mac1-regulated genes, the CatB catalase and the Sod4 superoxide dismutase encoding genes are those showing the highest induction next to *CTR3* (Fig. 5), suggesting Mac1-dependent induction of genes in low copper contributes to *Histoplasma* resistance to reactive oxygen. Even though we found that simultaneous loss of Ctr3 and CatB does not further attenuate virulence compared to loss of Ctr3 alone, a second catalase, CatP, is known to provide some redundancy with CatB (6). This second catalase was also upregulated by Mac1, albeit to a lesser degree (3.6-fold versus 2-fold for *CATB* versus *CATP*, respectively; Fig. 5). Unfortunately, we are not able to deplete both catalase functions to examine their contributions to *Histoplasma* virulence during adaptive immunity because their simultaneous loss attenuates *Histoplasma* virulence during the innate immune stage (6). The precise role of *Histoplasma* Sod4 is currently unknown, but the protein is homologous to iron/manganese-type superoxide dismutases, not copper-dependent superoxide dismutases, such as the extracellular Sod3 superoxide dismutase, which is essential for *Histoplasma* virulence (7). Nevertheless, the Mac1-dependent induction of three genes encoding non-copper-dependent enzymes important for reactive oxygen detoxification suggests that low copper sensed by Mac1 stimulates pathogenesis mechanisms beyond copper acquisition. We therefore propose that the copper restriction by macrophages following stimulation with IFN-γ during the adaptive immune response serves as a signal to *Histoplasma* yeasts of another IFN-γ-driven macrophage inflammatory response: enhanced production of ROS.

In addition to induction of catalases and a superoxide dismutase, Mac1 regulates expression of *ZRT1*, encoding a putative zinc transporter. This suggests that *Histoplasma* may use Mac1 sensing of decreased copper availability to regulate responses to another avenue of host defense, specifically zinc-sequestration-based nutritional immunity (28). Another zinc transporter, Zrt2, is important for virulence in *Histoplasma* (24), but the role of Zrt1 is unknown. However, Mac1 depletion did not result in increased sensitivity to the zinc chelator TPEN, possibly due to redundancy of Zrt1 with Zrt2. Iron limitation is another mechanism of nutritional immunity against fungi (29). Interestingly, copper limitation influences the expression of *Histoplasma* genes encoding enzymes for the production of iron-scavenging siderophores (23). However, while the expression of siderophore biosynthesis genes is regulated by copper, this is independent of Mac1 (Fig. 5). In contrast to Mac1-regulated genes that are induced in low copper, copper restriction reduces expression of siderophore-biosynthesis genes, at least *in vitro* (Fig. 5). Since copper becomes limited in the phagosome during the adaptive immune response, this may indicate that *Histoplasma* siderophore synthesis and thereby iron acquisition is reduced at this point during infection. However, this contrasts with the reported requirement for siderophores for *Histoplasma* proliferation during the adaptive immune response (23). We suspect that low-iron induction of siderophore biosynthesis genes through Sre1 (30) overcomes any minor copper regulation of this fungal gene cluster. Interestingly, inspection of the promoters of these Mac1-independent copper-regulated genes revealed multiple instances of a consistent DNA motif (TCTGCTC[A/G]), which is similar, but not identical to the published S. cerevisiae CuRE (data not shown). We speculate that this may represent regulation by the high-copper-sensing AceI transcription factor, which shares 55% identity and 70% similarity with Mac1 in the copper fist DNA-binding domain and could therefore have a similar binding motif. Given the limitations of transcription factor binding site prediction using the S. cerevisiae CuRE, future DNA binding experiments with *Histoplasma* Mac1 and AceI will be required to elucidate their true binding motifs.

Our results indicate that Mac1 coordinates multiple aspects of *Histoplasma* virulence beyond simple acquisition of copper. In low copper, Mac1 induces the expression of Ctr3, as well as CatB, CatP, and Sod4 antioxidant genes, none of which use copper as a cofactor or have obvious connections to copper. In addition, 10 other genes of unknown function are induced, including three putative transcription factors (Hca_02037, Hca_07432, and Hca_09066; Fig. 5). Together, these findings suggest that *Histoplasma* uses Mac1 sensing of a low-copper environment to signal residence within the phagosome of activated phagocytes. Within this environment, fungal mechanisms to combat nutritional immunity (i.e., copper and zinc restriction by the host) and to provide increased ROS detoxification are stimulated. While temperature is the primary regulator of *Histoplasma* virulence which enables colonization and proliferation within host cells during innate immune responses, sensing of copper limitation by Mac1 allows *Histoplasma* to further adapt to the enhanced antifungal defenses imposed by the host macrophage during the adaptive immune response.

## MATERIALS AND METHODS

### *H. capsulatum* strains and cultivation.

All Histoplasma capsulatum strains used in this study were derived from the G217B clinical isolate and are listed in Table S1A. Strains were cultivated using *Histoplasma-*macrophage medium (HMM; which contains 10 nM CuSO_4_) or with RPMI depleted of any trace metals through treatment with Chelex resin. Media were buffered to pH 5 and supplemented with FeSO_4_, ZnSO_4_, or CuSO_4_ where necessary. For growth on solid media, strains were plated on HMM solidified with 0.6% agarose supplemented with 25 μM FeSO_4_. For growth of uracil auxotrophs, HMM was supplemented with 100 μg/mL uracil. Yeast liquid cultures were grown at 37°C with continuous shaking (200 rpm). For growth experiments and infections, *Histoplasma* yeast cultures were grown to late exponential phase. Growth was quantified by measuring culture turbidity using optical density at 595 nm using a BioTek Synergy 2 plate reader or by enumeration of CFU via serial dilutions on solid HMM.

10.1128/mbio.03773-21.1 TABLE S1 (A) *Histoplasma* strains utilized in this study. (B) CTR3 promoter transcriptional reporter constructs. Download Table S1, DOCX file, 0.01 MB. Copyright © 2022 Ray and Rappleye. 2022 Ray and Rappleye. https://creativecommons.org/licenses/by/4.0/ This content is distributed under the terms of the Creative Commons Attribution 4.0 International license.

### Generation of RNAi and mutant lines.

To generate mutant *Histoplasma* strains, plasmids were introduced via an Agrobacterium tumefaciens delivery system and recovered using uracil selection (31). For *CTR3* promoter experiments, the G217B-derived strain WU15 was transformed using plasmids containing the manipulated promoter transcriptionally fused to a GFP reporter as outlined in Table S1B. For gene depletion experiments, *Histoplasma MAC1* and *CATB* were depleted from the WU15-derived GFP-fluorescent strain OSU194 using RNA interference (RNAi) (5). Strains OSU310 and OSU315 (uracil auxotrophic and prototrophic *ctr3* mutants, respectively) were isolated previously from an *Agrobacterium-*mediated mutagenesis screen (8).

### Identification of Mac1 binding motifs.

Putative Mac1 binding motifs were identified using the JASPAR nucleotide frequency matrix of the S. cerevisiae Mac1 copper response element (CuRE, MA0326.1). The nucleotide sequence corresponding to 1 kb upstream of the CTR3 coding sequence was extracted from the *Histoplasma* strain G217B genome and searched for instances of the S. cerevisiae CuRE using the FIMO program from the MEME suite (6). Results were restricted to a *P* value of <0.001. Putative binding motifs were then manually inspected for similarity to the S. cerevisiae Mac1 copper response element.

### *CTR3* promoter manipulation and measurement of response to copper concentration.

To determine the minimal *CTR3* promoter required for gene induction in response to low copper, the *CTR3* promoter was sequentially truncated and cloned upstream of a GFP fluorescent transcriptional reporter, then transformed into *Histoplasma.* For each construct, 16 transformants were randomly selected and spotted onto standard solid HMM (10 nM CuSO_4_) and HMM supplemented with 10 μM CuSO_4_. In addition, a strain expressing GFP driven by the constitutive *TEF1* promoter was included as a nonregulated GFP positive control, as well as the GFP negative strain G217B. Images were obtained for each colony using a modified transilluminator system with GFP emission filters (32), and the GFP fluorescence of each colony was quantified using Fiji software tools (33). GFP fluorescence in the *CTR3* promoter reporter strains was reported relative to control spots of the constitutively expressed GFP under the control of the *TEF1* promoter (*P_TEF1_-GFP*). To test the necessity of Mac1 binding motifs for the copper response of the *CTR3* promoter, the Mac1 binding motifs were scrambled individually or together using overlap PCR and then cloned into the same GFP expression vector used for the promoter truncations. Constructs were transformed into *Histoplasma* yeasts, isolates were plated in high- and low-copper media, and the GFP fluorescence was quantified as described above.

### Phylogenetic analysis of fungal copper-dependent transcription factors and identification of the *Histoplasma* MAC1 ortholog.

Protein sequences for copper-dependent transcription factors were retrieved from fungal genomes, including H. capsulatum strain G217B, by BLAST search using the copper-fist DNA-binding domain sequence (pfam00649), as well as the known copper-dependent DNA-binding proteins Cup2 (S. cerevisiae), Cuf1 (C. neoformans), Cuf2 (S. pombe), Mac1 (S. cerevisiae), and AceA (A. nidulans). Protein sequences were trimmed to the N-terminal 70 amino acids that includes the copper-fist DNA-binding domain. Sequences were aligned using CLUSTAL-Omega (34), and the alignment was used to construct a phylogenetic tree. Clades were identified and *Histoplasma* copper-fist proteins named using fungal orthologs with demonstrated (not predicted) functions.

### Determination of yeast sensitivity to metal ion limitation and toxicity.

*Histoplasma* yeasts from exponentially growing cultures were collected, washed, and resuspended in RPMI medium containing serial dilutions of the copper chelator bathocuproinedisulfonic acid (BCS), the iron chelator bathophenanthrolinedisulfonic acid (BPS), or the zinc chelator *N*,*N*,*N′*,*N′*-Tetrakis(2-pyridylmethyl)ethylenediamine (TPEN) or, alternatively, in HMM medium containing supplemental copper(II) sulfate. *Histoplasma* cultures were incubated at 37°C with continuous shaking (200 rpm), and yeast growth was quantified by measuring the culture turbidity (optical density at 595 nm) with a BioTek Synergy 2 plate reader. The *Histoplasma* growth in each medium is reported relative to the growth in basal medium alone.

### Quantification of extracellular catalase activity.

*Histoplasma* culture supernatants were collected from 5-day-old stationary-phase cultures grown in either standard HMM (10 nM CuSO_4_) or HMM supplemented with 5 μM CuSO_4_. After collection, BCS was added to a final concentration of 10 μM to chelate any trace copper that might interfere with catalase activity. To monitor CatB extracellular catalase activity, 15 mM H_2_O_2_ was added to culture supernatants, and the degradation of H_2_O_2_ was monitored by assaying samples at 0, 5, 10, 15, and 20 min with a cobalt/bicarbonate-based colorimetric indicator of peroxide [1.0 mg/mL Co(NO_3_)_2_·6H_2_O, 0.5 mg/mL (NaPO_3_)_6_, 81 mg/mL NaHCO_3_ in H_2_O]. The absorbance of the colorimetric indicator was measured at 440 nm, as described previously (35).

### Transcriptional analyses.

RNA used for transcriptional analyses was collected from log-phase wild-type and *MAC1*-RNAi *Histoplasma* yeast cultures grown in either HMM (low copper) or HMM with 5 μM added CuSO_4_ (high copper), with samples prepared from two biological replicates for each condition. Yeasts were collected via centrifugation (5 min at 2,000 × *g*), and RNA was released from cells by resuspension of yeasts in TRIzol reagent (Thermo Fisher) and mechanical disruption of yeasts with 0.5 mm-diameter glass beads. Cellular RNA was purified with Direct-zol RNA miniprep columns (Zymo Research). RNA samples were treated with Turbo DNase (Invitrogen) to remove contaminating DNA. For RT-qPCR, cDNA was generated from total RNA using Maxima reverse transcriptase (Thermo Scientific) primed with random pentadecamers. Quantitative PCR was performed using gene-specific primer pairs, and amplified products were quantified via SYBR green-based detection (SensiMix SYBR No-ROX kit; Bioline). After normalization to the *Histoplasma* actin (*ACT1*) gene, changes in gene expression relative to *TEF1* were determined using the cycle threshold (ΔΔ*C_T_*) method (36). For RNA-seq, RNA sample quality was assessed using a Qubit fluorometer (Invitrogen). mRNA libraries were generated by poly(A) selection, fragmentation, reverse transcription, and ligation to Illumina adapters. RNA-seq data were obtained from 150-bp paired-end reads using an Illumina HiSeq instrument (Genewiz). The quality of short reads was assessed and trimmed with FastQC (37). Reads were aligned to the *Histoplasma* G217B genome using STAR v2.5.0a (38), and reads mapping to each predicted coding region (4) were counted using HTseq v0.8.0 (39). Differentially expressed genes were identified using EdgeR v3.32.0 (40), and genes showing at least 2-fold differential expression were identified.

### Macrophage cell culture and infection.

A LacZ-expressing P388D1 murine macrophage cell line was utilized for all *in vitro* infection experiments. Macrophages were cultivated at 37°C with 5% CO_2_/95% air in Ham F-12 media (Corning) containing 10% fetal bovine serum (FBS; Sigma). Macrophages were infected with *Histoplasma* yeasts at a multiplicity of infection (MOI) of 1:1 (yeasts:macrophages) for intracellular yeast enumeration or an MOI of 1:3 for macrophage survival assays. Noninternalized yeasts were removed after 2 h by shaking plates (1 min at 1,000 rpm) and replacement of the medium on cells with fresh Ham F-12 with 10% FBS. Cultures of infected macrophages were mixed daily by shaking (1 min at 1,000 rpm) to disperse yeasts from lysed cells. For intracellular proliferation assays, macrophages were lysed with H_2_O and scraping of wells with a pipette tip. Serial dilutions of lysates were plated on solid HMM for enumeration of CFU. For macrophage survival assays, surviving macrophages were quantified at 0, 3, 4, and 5 days postinfection by measuring the LacZ activity relative to that of uninfected macrophages (41). To quantify LacZ activity, culture medium was replaced with a lysis solution comprised of 0.1% Triton X-100 with 1 mg/mL *o*-nitrophenyl-β-d-galactopyranoside (ONPG), and the β-galactosidase activity in each well was then measured using a BioTek Synergy 2 plate reader (absorbance at 420 nm with correction at 600 nm).

### Murine model of histoplasmosis.

Suspensions of 2 × 10^4^ *Histoplasma* yeast were introduced intranasally into isoflurane-anesthetized wild-type C57BL/6 mice. At 6, 12, or 14 days postinfection, mice were euthanized, and lungs (and spleens when indicated) were collected, homogenized in HMM, and then serially diluted and plated onto solid HMM for CFU enumeration. Animal experiments were performed in compliance with the National Research Council’s *Guide for the Care and Use of Laboratory Animals* and were approved by the Institutional Animal Care and Use Committee (IACUC) at The Ohio State University (protocol 2007A0241).

### Statistical analyses.

Data were analyzed by Student’s t-test (Prism v9; GraphPad Software) to determine statistically significant differences, which are indicated in the figure graphs by asterisks (*, *P < *0.05; **, *P < *0.01; ***, *P < *0.001; ****, *P* < 0.0001).

### Data availability.

RNAseq data has been submitted under BioProject ID: PRJNA812927. All strains are available from the authors upon request.

10.1128/mbio.03773-21.2 TABLE S2 Primers used in this study. Download Table S2, DOCX file, 0.01 MB. Copyright © 2022 Ray and Rappleye. 2022 Ray and Rappleye. https://creativecommons.org/licenses/by/4.0/ This content is distributed under the terms of the Creative Commons Attribution 4.0 International license.
