# Supplementary material for: Mac1-Dependent Copper Sensing Promotes Histoplasma Adaptation to the Phagosome during Adaptive Immunity
Source: mBio. 2022 Apr 11;13(2):e03773-21. doi: 10.1128/mbio.03773-21 (PMC9040751; doi:10.1128/mbio.03773-21)
Supplement: TABLE S1 [file mbio.03773-21-st001.docx]

**Table S1A. *Histoplasma* strains utilized in this study**

| **Strain^a^** | **Genotype^b^** |
| --- | --- |
| G217B | wild type clinical isolate (ATCC 26032) |
| WU15 | *ura5-42Δ* |
| OSU310 | *ura5-42Δ zzz::pQS01 (apt3, RFP) ctr3-2::pBHt2 (hph)* |
| OSU315 | *ura5-42Δ zzz::pQS01 (apt3, RFP) ctr3-2::pBHt2(hph) zzz::pCR628(URA5, GFP)* |
| OSU374 | *ura5-42Δ zzz::pAG21 (apt3^R^, GFP) zzz:pED02 (URA5, gfp-RNAi)* |
| OSU376 | *ura5-42Δ zzz::pAG21 (apt3^R^, GFP) zzz::pSR25 (URA5, gfp:MAC1-RNAi)* |
| OSU430 | *ura5-42Δ zzz::pAG21 (apt3^R^, GFP) zzz::pJX04 (URA5,gfp:CATB-RNAi)* |
| OSU431 | *ura5-42Δ zzz::pQS01 (apt3, RFP) zzz::pED02 (URA5, gfp-RNAi)* |
| OSU432 | *ura5-42Δ zzz::pQS01 (apt3, RFP) zzz::pJX04 (URA5, gfp:CATB-RNAi)* |
| OSU433 | *ura5-42Δ zzz::pQS01 (apt3, RFP) ctr3-2::pBHt2 (hph) zzz::pED02 (URA5,gfp-RNAi)* |
| OSU434 | *ura5-42Δ zzz::pQS01 (apt3, RFP) ctr3-2::pBHt2 (hph) zzz::pJX04 (URA5, gfp:CATB-RNAi)* |

^a^ OSU310 and OSU315 from (Shen Q, et al. 2018) or strains were derived from OSU194 (Garfoot AL, et al., 2016) or OSU233 (Shen Q, et al., 2018)

^b^ gene designations:

*apt3*: aminoglycoside phosphotransferase (G418 resistance)

*CATB*: extracellular catalase

*CTR3*: copper transporter

*GFP*: green fluorescence protein

*hph*: hygromycin phosphotransferase (hygromycin B resistance)

*MAC1*: metal-binding activator

*RFP*: red-fluorescence protein (td-tomato)

*URA5*: orotate phosphoribosyltransferase

**Table S1B. CTR3 promoter transcriptional reporter constructs**

| **Plasmid^a^** | **Promoter-GFP fusion** |
| --- | --- |
| pCR639 | *TEF1* (654bp upstream of the CDS) |
| pSR28 | *CTR3* (485bp upstream of the CDS) |
| pSR29 | *CTR3* (285bp upstream of the CDS) |
| pSR32 | *CTR3* (335bp upstream of the CDS) |
| pSR33 | *CTR3* (166bp upstream of the CDS) |
| pSR36 | *CTR3* (335bp upstream of the CDS, scrambled CuRE C) |
| pSR37 | *CTR3* (335bp upstream of the CDS, scrambled CuRE B) |
| pSR38 | *CTR3* (335bp upstream of the CDS, scrambled CuRE B and C) |
| pSR42 | *CTR3* (335bp upstream of the CDS, scrambled CuRE A) |
| pSR43 | *CTR3* (335bp upstream of the CDS, scrambled CuRE A and B) |
| pSR44 | *CTR3* (335bp upstream of the CDS, scrambled CuRE A and C) |
| pSR45 | *CTR3* (335bp upstream of the CDS, scrambled CuRE A, B, and C) |

^a^ All constructs contained the *URA5* gene for selection and were transformed into the non-fluorescent strain WU15
