# Supplementary material for: Mac1-Dependent Copper Sensing Promotes Histoplasma Adaptation to the Phagosome during Adaptive Immunity
Source: mBio. 2022 Apr 11;13(2):e03773-21. doi: 10.1128/mbio.03773-21 (PMC9040751; doi:10.1128/mbio.03773-21)
Supplement: TABLE S2 [file mbio.03773-21-st002.docx]

**Table S2. Primers used in this study**

| Primer | Primer sequence (5' to 3') | Direction^a^ |
| --- | --- | --- |
| ACT1-5 | GGTTTCGCTGGCGATGATGCTC | forward |
| ACT1-9 | AAGGACGGCCTGGATGGAGACG | reverse |
| CATB-5 | AGGCGCGCCCGTCCACACCTTCCGACTTG | forward |
| CATB-6 | CACTCGAGATCCAACGGGAACCGATTCAG | reverse |
| CATB-7 | GCACTAGTCGTCCACACCTTCCGACTTG | forward |
| CATB-8 | AAACCGGTATCCAACGGGAACCGATTCAG | reverse |
| CTR1-1 | GCCGCTGAACCGTACACCGCCCT | forward |
| CTR1-2 | AATGGGTTTCTGAACTGCCGAAT | reverse |
| CTR-2 | AGGCGCGCCTTTGATTTTAGATTGGCAGAGCAGTCAAC | reverse |
| CTR2-1 | GTGCTCCTGACTGCTGGCTACGA | forward |
| CTR2-2 | GAAAGAAGAGGGGCATTGGGAGT | reverse |
| CTR3-12 | GATGCCTAGGTAGGGATGCCAAC | forward |
| CTR3-13 | CGCTCGAGCCACAACATACGGTCACCT | reverse |
| CTR3-22 | GCGGATCCGACCCTGAGCGAAGATTTGAGC | forward |
| CTR3-23 | GCGGATCCGGCGGAGGACACTGACAACG | forward |
| CTR3-24 | GCGGATCCGTTCCTGAGTTCCTGTAGAGTTTGAC | forward |
| CTR3-25 | GCGGATCCTAAACTCACTTACCTCCAGTCATAAC | forward |
| CTR3-28 | CGAAGAACGTTCTTAATTAACAACCTAAATAAATA | forward |
| CTR3-29 | GTTAATTAAGAACGTTCTTCGCTCAGGGTCA | reverse |
| CTR3-30 | AGTATGGATTCGTAATAAACTCACTTACCTCCAGTC | forward |
| CTR3-31 | GTTTATTACGAATCCATACTTTAAGATAGCCCTAG | reverse |
| CTR3-36 | TTGACCCGAGAACTGGATTTGAGCGAATTAACAA | forward |
| CTR3-37 | TTGACCCGAGAACTGGAACGTTCTTAATTAACAA | forward |
| CTR3-38 | TCCAGTTCTCGGGTCAAAGTCTAACGCGGA | reverse |
| CTR3-45 | GCGGATCCAATAGATAAATATAATATAAAGG | forward |
| CTR3-46 | GCGGATCCTCACTACTTGATGCTCGAAC | forward |
| GFP-40 | CACGCCGTAGGTCAGGGTGGTC |  |
| MAC1-1 | TATGGCGCGCCGCAACGGGAATAATGGAGCC | forward |
| MAC1-2 | CAGCTCGAGTAGGGATGAGAAGCACAGCC | reverse |
| MAC1-3 | GCGACTAGTGCAACGGGAATAATGGAGCC | forward |
| MAC1-4 | GCGACGTCGTAGGGATGAGAAGCACAGCC | reverse |
| RB-12 | GGAAACGACAATCTGATCCAAGC |  |
| TEF1-8 | GCTCTGCTTGCTTTCACCCTTG | forward |
| TEF1-9 | TCTCCTTGTTCCAGCCCTTGT | reverse |
| TUB2-3 | GCCTCGTACGCTCGCTTCGC |  |

^a^ Direction relative to gene transcription
